# Single‐cell RNA sequencing analysis reveals transcriptional heterogeneity of multiple primary lung cancer

**DOI:** 10.1002/ctm2.1453

**Published:** 2023-10-17

**Authors:** Wei Guo, Bolun Zhou, Fenglong Bie, Qilin Huai, Xuemin Xue, Lei Guo, Fengwei Tan, Qi Xue, Liang Zhao, Shugeng Gao

**Affiliations:** ^1^ Department of Thoracic Surgery National Cancer Center/National Clinical Research Center for Cancer/Cancer Hospital Chinese Academy of Medical Sciences and Peking Union Medical College Beijing P. R. China; ^2^ Key Laboratory of Minimally Invasive Therapy Research for Lung Cancer Chinese Academy of Medical Sciences Beijing P. R. China; ^3^ Department of Thoracic Surgery Shandong Provincial Hospital Affiliated to Shandong First Medical University Jinan Shandong P. R. China; ^4^ Department of Pathology National Cancer Center/National Clinical Research Center for Cancer/Cancer Hospital Chinese Academy of Medical Sciences and Peking Union Medical College Beijing P. R. China

**Keywords:** heterogeneity, multiple primary lung cancer, single‐cell RNA sequencing, tumour microenvironment

## Abstract

**Introduction:**

With the advancements in early diagnosis, more and more patients with multiple primary lung cancer (MPLC) have been identified. However, the progression of MPLC involves complex changes in cell composition and metabolic function, which remains largely controversial.

**Objective:**

Our study aims to comprehensively reveal the cellular characteristics and inter‐cellular connections of MPLC.

**Methods:**

We performed scRNA‐seq from 23 samples of six MPLC patients, combined with bulk whole‐exome sequencing. We performed trajectory analysis to investigate the transition of different cell types during the development of MPLC.

**Results:**

A total of 1 67 397 cells were sequenced derived from tumour and adjacent tissues of MPLC patients, and tumour, normal, immune and stromal cells were identified. Two states of epithelial cells were identified, which were associated with immune response and cell death, respectively. Furthermore, both CD8^+^ naïve and memory T cells participated in the differentiation of CD8^+^ T cells. The terminal states of CD8^+^ T cells were exhausted T cells and cytotoxic T cells, which positively regulated cell death and were implicated in the regulation of cytokine production, respectively. Two main subpopulations of B cells with distinct functions were identified, which participate in the regulation of the immune response and antigen presentation, respectively. In addition, we found a specific type of endothelial cells that were abundant in tumour samples, with an increasing trend from normal to tumour samples.

**Conclusions:**

Our study showed the comprehensive landscape of different cells of MPLC, which might reveal the key cellular mechanisms and, therefore, may provide new insights into the early diagnosis and treatment of MPLC.

## Graphical abstract

- The cellular characteristics and intercellular connections of MPLC are presented.
- The dynamic changes of cellular composition from normal samples to AIS/MIA and finally to IAC in MPLC are demonstrated.
- Two states of malignant cells with different functions, and various novel subpopulations of immune and stromal cells are determined in MPLC patients.

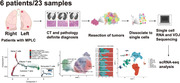

## 1 INTRODUCTION

Globally, lung cancer is regarded as the main cause of cancer‐related mortality.^1^, ^2^, ^3^, ^4^ The occurrence of two or more primary lung cancers in the same individual is known as multiple primary lung cancer (MPLC).^5^ Port et al. reported that 16% of patients with resectable stage I‐III non‐small cell lung cancer (NSCLC) have MPLC.^6^ Concomitant with the rising prevalence of lung cancer, the incidence of MPLC is also increasing.^5^, ^7^ Thus, an in‐depth understanding of its mechanism and novel therapeutic strategies for MPLC are currently needed.

Tumour heterogeneity describes differences between tumours of the same apparent type but with different carcinogen exposures and genetic backgrounds (inter‐tumoural heterogeneity) or differences between cancer cells within the same tumour (intra‐tumoural heterogeneity).^8^, ^9^, ^10^, ^11^ MPLC, by definition, arises in various parts of the lung parenchyma in the same individual and shares an identical environmental exposure profile and genetic background.^12^ According to recent publications, differences were identified in genomic profiles between different MPLC lesions including mutational spectra, chromosomal structural variations, copy number aberrations and somatic point mutations.^12^, ^13^, ^14^ In addition to malignant cells, the very complex and varied tumour ecosystem also involves host cells that interact with the tumour including stromal fibroblasts, endothelial cells and a range of immune cells that regulate tumour development and metastasis.^15^, ^16^ Although a great number of clinical trials investigating immune checkpoint inhibitors have been conducted, the heterogeneity of cell types in the tumour microenvironment (TME) in MPLC remains unclear.^17^, ^18^ Decoding the intricate interaction between tumour cells and the TME in MPLC is, therefore, crucial.

In the present study, we performed single‐cell RNA sequencing (scRNA‐seq) and whole‐exome sequencing (WES) on 17 tumour samples and 6 matched normal samples from 6 patients with MPLCs. By comparing tumours at different stages in MPLCs and adjacent tissues, we characterized the transcriptome features of stromal cells, immune cells and malignant cells of MPLCs. We unraveled dynamic shifts in inter‐cellular connections, variety of cell subtypes and cell percentage, revealing novel information about the molecular underpinnings of MPLC and LUAD development.

## 2 MATERIALS AND METHODS

### 2.1 Sample collection

MPLC patients with two or three tumours in the ipsilateral or bilateral lungs and who underwent surgery at our hospital were enrolled. After MPLC samples were resected, tumour tissues were cut and processed for pathological diagnosis, scRNA‐seq and WES. The clinical characteristics and specimen information of these patients are presented in Supporting Information Table S1. This study was approved by the Ethics Committee of our institution.

### 2.2 Tissue dissociation

We incubated the samples at 37°C for 30−60 min in an Eppendorf thermomixer at 700 rpm. After incubation, we filtered the cell suspension through a pre‐wetted 70 μm MACS SmartStrainer, placed in 50 mL tubes and centrifuged at 300 *g* for 5 min at 4°C. And the cell pellet was resuspended in 1 mL of chilled 1X PBS with 0.04% BSA buffer, and a Countess II automated cell counter was used to count the cell concentration and viability. If the percentage of viable cells was less than 70%, dead cells were removed to increase the proportion of viable cells. An appropriate volume of buffer was added to the cell suspension and gently mixed to achieve a target cell concentration of 700−1200 cells/μL. Once the target cell concentration was obtained, GEM generation was immediately performed with 10X Genomics reagents. The remaining cells were frozen at −80°C for WES (not more than 5 × 10^6^ cells were used).

### 2.3 Whole‐exome sequencing and analysis

We used a QIAamp DNA Mini Kit (Catalog# 51304) to process DNA extraction. The cell pellet was resuspended in PBS to a final volume of 200 μL, and 20 μL proteinase K was added to each sample and mixed by vortexing. Then, 4 μL of RNase A stock solution (100 mg/mL) was added to the sample and mixed completely. We placed the QIAamp Mini Column in a clean 2 mL collection tube containing the discarded filtrate. And we carefully opened the column and added 500 μL buffer AW1. Then we closed the cap and centrifuged the tube at 6000 x *g* for 1 min. This step was repeated with buffer AW2 and eluted with 100 μL buffer AE. One microliter of DNA sample was taken for quantification on a 1% agarose gel. After passing the quality control (QC), the SureSelect XT Library Prep Kit (Catalog#: 5500‐0132) was used for library construction and QC according to the official protocol. Finally, we used an Illumina Nova 6000 for 150 bp paired end (PE 150) sequencing.

Read quality was calculated for all samples by using FastQC (version 0.11.2)^19^ software with the default parameters. We used the BWA‐MEM^20^ algorithm to compare the sequencing data to the reference genome (GRCh38), obtain the bam file, mark the PCR reads in the bam file, rearrange the regions that may have indel mutations and recalibrate the quality of each base pair. In this analysis, the Sentieon (201611.02)^21^ workflow was carried out to detect SNVs and indels. The tumour mutation burden (TMB) result was the sum of non‐synonymous mutations and indel numbers in the exon region divided by the length of the total exon. The variant allele frequency (VAF) means the coverage depth of reads that support alternate and mutant alleles at a particular site in the genome, which is the proportion of the total coverage depth of reads. VAF is commonly reported and may aid in discerning whether a variant is germline or somatic.

### 2.4 Chromium 10× genomics library and sequencing

For 10X chromium single‐cell immune profiling, target tissue was prepared into single‐cell suspensions by using commercial enzymes from Miltenyi. Then, we used a Countess II Automated Cell Counter to determine cell concentration and viability. The cells were diluted to 700−1200 cells/μL. Cell loading, GEM generation and library construction of T cells and B cells were performed based on the Chromium Next GEM Single‐Cell 5′ Kit official protocol. After library construction, we used an Agilent 2100 Bioanalyzer to perform a library check. Finally, we used an Illumina Nova 6000 for PE 150 sequencing. We performed additional sequencing to obtain coverage of at least 50K reads per cell.

### 2.5 Single‐cell RNA sequencing data alignment

The 10× Genomics single‐cell raw sequencing paired‐end reads were processed using Cell Ranger (version 5.0.1) and the 10× human genome GRCh38 as the reference.^22^ The output‐filtered gene expression matrices were analyzed by the Seurat R package (version 4.0.0). Low‐quality cells with (i) less than 500 unique molecular identifiers (UMIs); (ii) more than 25% UMIs derived from the mitochondrial genome; or (iii) more than 5000 or less than 200 genes detected (genes expressed at a proportion > 0.1%) were removed. The FindVariableFeatures function was used to select 2000 features with high cell‐to‐cell variation. Finally, with the use of FindClusters and FindNeighbors functions, we clustered cells using the suitable PCs to calculate the Uniform Manifold Approximation and Projection (UMAP) for dimension reduction. We used the RunUMAP function with default settings to perform non‐linear dimensional reduction.

### 2.6 Cell type annotation

After non‐linear dimensional reduction, cells could be clustered together according to common features. UMAP was used to project all cells into two‐dimensional space. We used the FindAllMarkers function to find the marker genes with significant differences in expression for each cluster. All clusters were classified and annotated based on these significantly different canonical markers.^23^ We classified clusters expressing no canonical cell type markers as ‘Others’.

### 2.7 Identification of malignant epithelial cells

The R code inferCNV (https://github.com/broadinstitute/infercnv) was used to infer the copy number variations (CNVs) in epithelial cells from the scRNA‐seq data. Putative non‐malignant cells were epithelial cells (EPCAM^+^) of the normal sample and we used their CNV estimates to determine a baseline. The epithelial cells from tumour samples were compared to the normal reference baseline. The CNV *R*‐scores in different regions are readily apparent and are overabundant or less abundant in the regions of the tumour genome compared to those of putative non‐malignant cells.

The mean square of the CNV estimates across all genomic sites served as the definition for the estimated CNV signal. The Pearson correlation coefficient between the typical CNV pattern of the top 5% of cells and CNV pattern of each cell from the same tumour in terms of CNV signal was used to calculate the CNV *R*‐scores.^24^ Epithelial cells (EPCAM^+^) with CNV R‐scores ≥0.4 and CNV signal ≥0.03 were defined as malignant cells, while those with CNV *R*‐scores ≤0.4 and CNV signal ≤0.03 were defined as non‐malignant cells. Cells that did not meet either of the above two conditions were defined as unresolved cells.

### 2.8 CellPhoneDB

CellPhoneDB was used with default parameters to infer the interactions between the different kinds of cells on a linear transformation‐scaled data matrix. According to the expression of a receptor from one cell type and a ligand from another, the approach predicts probable receptor‐ligand interactions.^25^ We only took into account the receptors and ligands that were expressed by more than 30% of the cells in the particular cluster. We used Cytoscape (version 3.8.2) to perform network visualization (version 3.8.2).

### 2.9 Trajectory analysis

We used Monocle (version 2.22.0) to conduct the trajectory analysis.^26^ We ordered the subclusters of cells, such as T cells or epithelial cells, onto a trajectory based on the union of highly variable genes obtained from the cells of the subcluster, which were from normal, minimally invasive adenocarcinoma (MIA) and invasive adenocarcinoma (IAC) samples. All parameters of Monocle2 were set to the defaults (cut‐off criteria: mean expression > 0.001; dispersion_empirical > dispersion_fit). The orderCells function and DDRTree method were used to perform dimensional reduction and cell ordering.

### 2.10 Data processing of single‐cell VDJ libraries

We used the GRCh38 reference genome to align T‐cell receptor (TCR) and B‐cell receptor (BCR) reads, and Cellranger vdj (version 5.0.1) with default parameters to perform consensus *VDJ* annotation. The *R* library Immunarch (version 0.6.7) was used to calculate the distribution of the *V* gene clonotype and calculate statistics for some related indicators including overlap and correlation.

### 2.11 Statistical analyses

The statistical tools, methods and thresholds used for each analysis are specifically stated within Section 3 or explained in Section 2.

## 3 RESULTS

### 3.1 Comprehensive cellular landscape in MPLC

**FIGURE 1:**
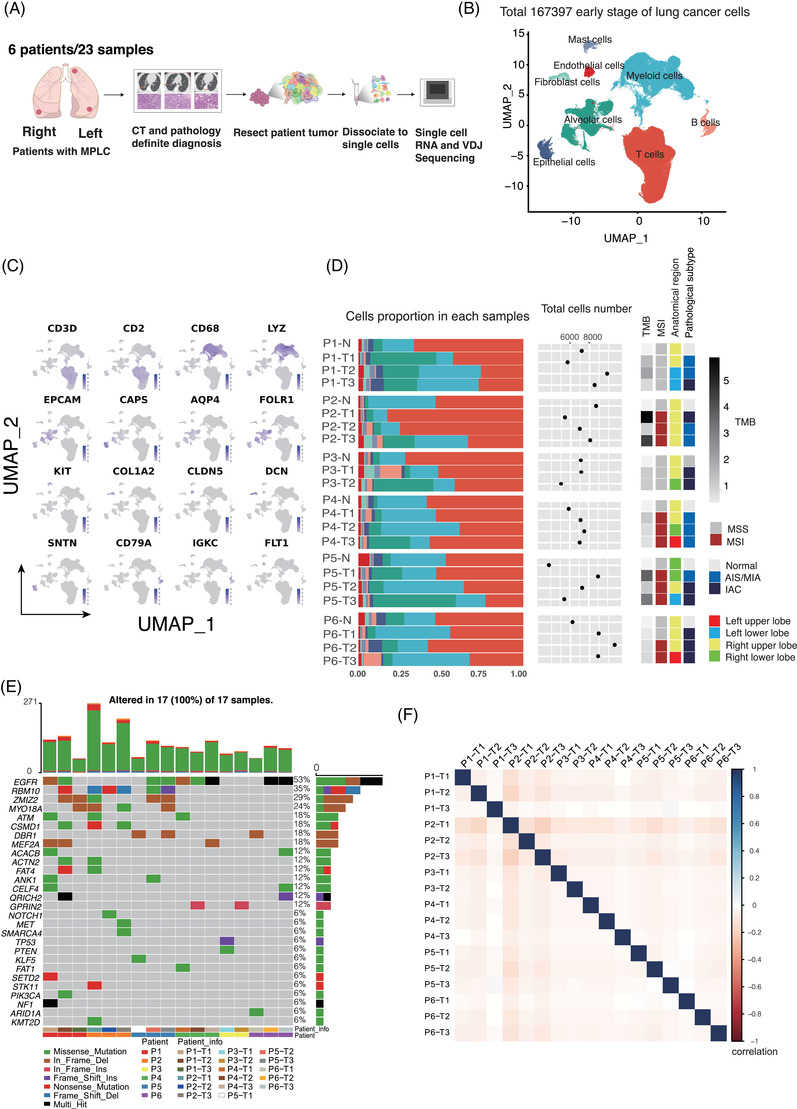
Cell‐type identification in MPLC using a combination of AIS/MIA, IAC and normal lung cells. (A) Workflow showing the scRNA‐seq experimental design and initial data exploration. (B) UMAP plot of 1 67 397 cells in the MPLC dataset. It consists of 12 124 malignant cells, 60 032 non‐malignant cells and 9 792 unresolved cells. (C) Marker genes were used to label clusters by cell identity as represented in the UMAP plot. (D) Average proportions of all kinds of cells among all normal, AIS/MIA and IAC samples. Proportions of global cell types in MPLC tissues and normal tissues in individual samples (bottom left). Cell number (bottom middle), TMB, MSI, anatomical region and infiltration in each sample (bottom right). (E) Mutational landscape of MPLC (n = 17). Genes with mutation frequencies of more than 5% and previously reported as significantly mutated genes in MPLC are shown for each region of the individual patient. (F) The correlation of the variant allele frequency detected by Senteion for the individual focus.

To elucidate the cellular development in MPLC, we analyzed a total of 1 67 397 cells from 23 sampling sites in 6 patients with multiple foci of early‐stage lung cancer from our institution (Figure 1A,B, Supporting Information Figure S1A,D and Table S2). Among these cells, 62 714 (23.2%) were from adenocarcinoma in situ (AIS) or MIA, 65 844 (37.5%) were from IAC and 38 839 (39.3%) were from normal lung tissues. A total of eight main cell types were determined in these un‐batched and comparable datasets by graph‐based UMAP (Figure 1B, Supporting Information Figure S1A,D). The marker‐based annotation correlated well with the cell types obtained via Cell Marker and SingleR version 1.0^27^, ^28^ (Figure 1B and Supporting Information Figure S1C). T cells and epithelial cells show great differences over the course of the development of MPLC. The proportion of B cells increased from normal lung tissues to AIS/MIA and IAC, whereas T‐cell abundance decreased (Figure 1D and Supporting Information Figure S1B).

The results of the genomic analysis revealed that the most frequently mutated genes were EGFR (53%), RBM10 (35%), ZMIZ2 (29%) and MYO18A (24%) (Figure 1E, Supporting Information Figure S1G and Data 1). Compared with NSCLC, MPLC has a distinct genetic mutation feature and the investigation of some prevalent mutations was currently lacking such as mutations in ZMIZ2 and MYO18A. The most frequent variant type was missense mutation (Supporting Information Figure S1G). The *C* > *A* and *C* > *T* transversions were enriched in these patients (Supporting Information Figure S1F). The correlation of the VAF for each individual focus was not significant (Figure 1F). And we found that lesions in each patient have different CNVs (Supporting Information Figure S1G). The significant mutations show that the foci in MPLC were independent and do not share an origin. Our findings revealed that some mutated genes were identified in both lesions of a single patient, whereas most mutations were unique in each lesion, indicating the genomic heterogeneity of different lesions of MPLC (Supporting Information Figure S1H).

### 3.2 Signatures and metabolic disturbances of malignant cells in MPLC

**FIGURE 2:**
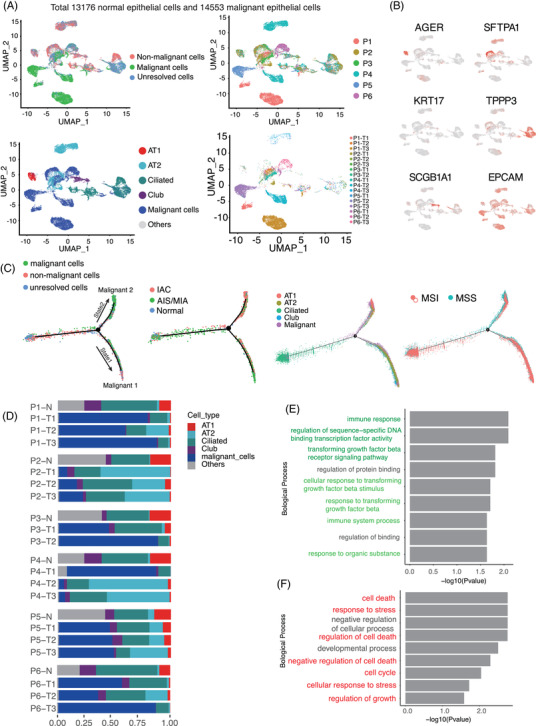
Identification and characterization of malignant and non‐malignant cells in normal and tumour EPCAM^+^ cells. (A) UMAP plot of all epithelial cells of the patients, color‐coded according to malignancy (upper left), cell type (lower left) or patient and sample information (right). (B) Canonical marker genes were used to label epithelial subtypes as represented in the UMAP plot. (C) Unsupervised pseudo‐time analysis of all epithelial cells visualizing information about malignancy, anatomical pathology, cell type and MSI. (D) Relative proportions of all epithelial cell subpopulations. (E, F) The enriched Gene Ontology terms for all signature genes in epithelial pseudo‐time analysis state 1 (top) and state 2 (bottom). The significant features are marked with colours.

A total of 13 176 normal epithelial cells and 14 553 malignant epithelial cells were obtained, and large‐scale CNVs were inferred with normal lung cells as references.^29^, ^30^ The CNV patterns showed that malignant cells formed clusters in patients with different origins, implying a high degree of inter‐tumoural heterogeneity (Figure 2A, Supporting Information Figure S2C,E). Alveolar cells are epithelial cells in the lung and play a pivotal role in maintaining the integrity and function of the alveoli. Both alveolar type I cells and alveolar type II cells were positive for EPCAM gene expression.^31^, ^32^ Other EPCAM^+^ cells were categorized as epithelial cells. We clustered the normal epithelial cells as ciliated airway epithelial cells, secretory club cells, alveolar type I cells, alveolar type II cells and other cells (Figure 2A,B, Supporting Information Figure S2A,D).^24^, ^33^ The relative abundance of AT2 cells was decreased in malignant samples compared with normal lung samples, and the fraction of AT1 cells increased from normal lung to AIS/MIA and IAC (Figure 2D).

In the pseudo‐time model, the upper‐ and the lower‐right branches were assigned as malignant cells mainly from IAC tissue, and two states in the evolution of malignant cells were identified from AIS/MIA to IACs (Figure 2C). Intriguingly, this trajectory began from non‐malignant AIS/MIA cells and branched in two malignant directions: State 1 was associated with immune response and some State 2 was associated with cell death in IAC (Supporting Information Figure S2G). Functional gene set enrichment analysis showed that the transition from State 1 was involved in the immune response, immune system process and response to transforming growth factor (Figure 2E). Shenoy et al. demonstrated that lung epithelial cells have the potential to affect the function of CD4^+^ cells and regulate barrier immunity, a finding congruent with our observations of State 1 epithelial cells.^34^ In addition, the expression of genes implicated in cell death, response to stress and cell cycle increased in State 2 (Figure 2F).

### 3.3 Clonal dynamics and phenotypic transitions of tumour‐infiltrating T cells in MPLC

**FIGURE 3:**
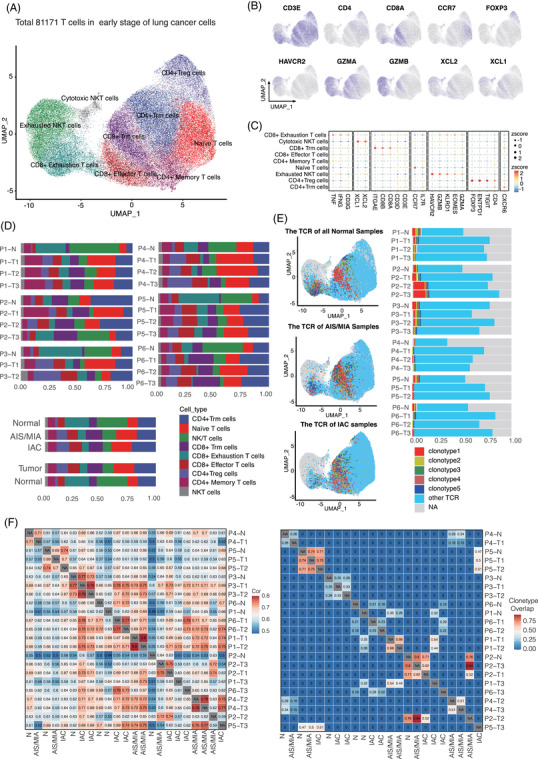
Clonal dynamics and phenotypic transitions of tumour‐infiltrating T cells in MPLC. (A) UMAP plot of a total of 81 171 T cells, coloured according to cell subtype. (B) Canonical marker genes were used to label T‐cells subtypes as represented in the UMAP plot. (C) The balloon plot of marker genes in the subtype of T cells. (D) Relative proportions of all T‐cells subpopulation. (E) UMAP of normal (upper), AIS/MIA (middle) and IAC (down) tumour‐infiltrating in T cells coloured by selected TCR clones. The bar plot (right) revealing slight differences in TCR clonotypes among each patient. (F) Overlap between T‐cell clones in different organ and cell types, divided by mouse. Each tile represents the overlap coefficient of clones. Colour intensity indicates overlap strength.

A total of 81 971 T cells were obtained from all samples and clustered into 10 subtypes. Two subtypes of CD4^+^ T cells were further clustered as CD4^+^ Treg cells and CD4^+^ Trm cells. Three subtypes of CD8^+^ T cells were further clustered as CD8^+^ exhausted T cells, CD8^+^ effector T cells and CD8^+^ Trm cells. One subtype of both CD4^−^ and CD8^−^‐positive T cells was further clustered as CD4^+^ memory T cells, one subtype of CD4^+^ or CD8^+^ T cells was clustered as naïve T cells, and two subtypes with no CD4 or CD8 expression were further clustered as exhausted NKT cells and cytotoxic NKT cells (Figure 3A‐C, Supporting Information Figure S3A,B).^35^, ^36^, ^37^, ^38^, ^39^ The relative abundances of classified CD4^+^ Treg cells and CD4^+^ Trm cells increased from normal lung tissues to AIS/MIA and IAC, while the proportion of CD8^+^ exhausted T cells decreased (Figure 3D). Next, we found that cells in normal and IAC samples shared more common clonotypes in TCRs than AIS/MIA group cells (Figure 3E, Supporting Information Figure S4A). And the differences in TCR clonotypes among each patient were slight (Figure 3E). The CD4^+^ Trm and naïve T cells shared more significant clonotypes with other T cells, and the shared clonotypes increased with the development of infiltration in the tumour tissue, suggesting an increasing transition from CD4^+^ Trm cells and naïve T cells to other activated T‐cell states (Supporting Information Figure 4A). The T‐cell phenotypes in AIS/MIA samples included more clonotype 1 than other normal and IAC samples, for which TRB‐TRA was ‘CASSGLAAKPGELF‐CAVRRGQNFV’ (Supporting Information Figure S4B). This sequence may play an important role in antigen presentation during tumour invasion. Compared with AIS/MIA samples, antigen from tumours to stimulate T cells was insufficient, and the expression of the related antigen presentation factor was low. As for the IAC tissue, the tumour has experienced stress from T cells in AIS/MIA, and new subclone evolution may not cause the same antigen stimulation. Therefore, clonotype 1, in which TRB‐TRA was ‘CASSGLAAKPGELF‐CAVRRGQNFV’, was significantly expressed in AIS/MIA samples (Figure 3F).

### 3.4 Trajectory profiling reveals the branched progression of T cells in MPLC

**FIGURE 4:**
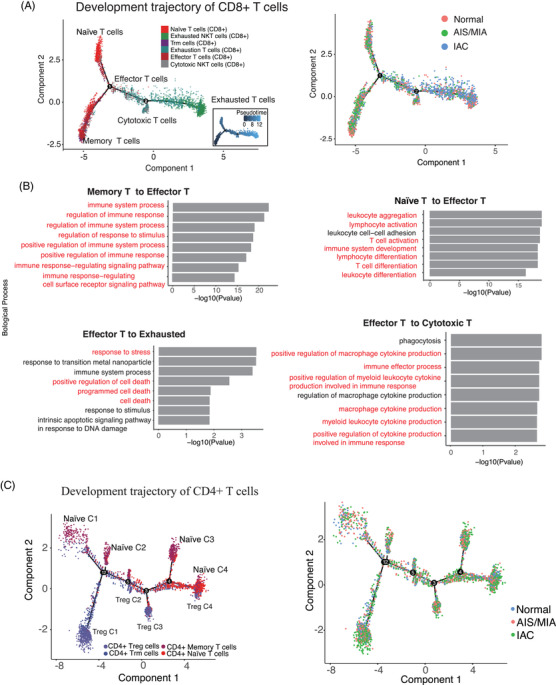
Trajectory profiling reveals the branched progression of T cells in MPLC. (A) Single‐cell trajectories of malignant cells in CD8^+^ T cells. The subplot coloured by global UMAP cluster (left) and anatomical pathology info (right). (B) The enriched gene ontology terms for all signature genes in CD8^+^ T cells pseudo‐time analysis in different states (Immune response [left‐up], T‐cell activation and differentiation [right up], response to stress and cell death [left‐down] and cytokine production [right down]) and the significantly features are marked with colours. (C) Single‐cell trajectories of malignant cells in CD4^+^ T cells coloured by global UMAP cluster and anatomical pathology info (down).

The results of trajectory analysis indicated that CD8^+^ naïve and memory T cells may differentiate into effector T cells (Figure 4A). We found that cells on the memory T to effector T branch expressed immune response markers, while naïve T‐cells transitioning to effector T cells expressed lymphocyte activation and T‐cell differentiation markers (Supporting Information Figure S3C, D). Cells on the effector T to exhausted T branch expressed responses to stress and cell death markers, while effector T cells expressed cytokine production markers (Supporting Information Figure S3C,D). Furthermore, Gene Ontology enrichment analysis revealed that the transition from memory T cells to effector T cells was mainly a process of immune response and regulation (Figure 4B). And the results of trajectory analysis indicated that CD4^+^ naïve and CD4^+^ Treg cells have four final differentiation states in themselves (Figure 4C, Supporting Information Figure S5A,B). Pathway analyses showed that the function of T cells in the Naïve_C2 cluster was closer to that of effector or exhausted T cells, while the function of the Naïve_C4 cluster was more closely related to that of other naïve clusters (Supporting Information Figure S5C [Left]). Moreover, Treg_C3 cells were enriched in the regulation of myeloid cell differentiation, while Treg_C2 cells mainly participated in the activation of TCR signalling and the regulation of cell differentiation pathways (Supporting Information Figure S5C [Right]).

### 3.5 B cells have two functional distribution subtypes in MPLC

**FIGURE 5:**
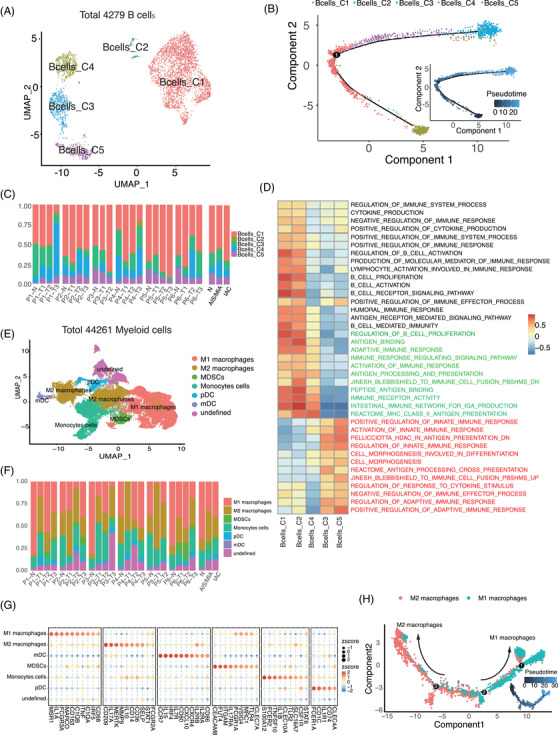
(A) Transition of the five main subtypes. (B) Unsupervised pseudo‐time analysis of all B cells. (C) Relative proportions of all B‐cell subpopulations. Each bar plot represents different samples, and the last bar plot is grouped by sample type (normal, AIS/MIA or IAC). (D) Differentially expressed pathways were scored per cell by GSVA between five B‐cell subtypes. (E) UMAP projection of 44 261 myeloid cells, showing the composition of the seven main subtypes. (F) Percentages of each myeloid cell subtype among normal, AIS/MIA and IAC samples. *Y* axis: Average percentage of samples across the three groups. Groups are separated by blank space. Each bar plot represents different samples, and the last bar plot is grouped by sample type (normal, AIS/MIA and IAC). (G) Balloon plot of marker genes in the myeloid cell subtype. (H) Unsupervised pseudo‐time analysis of all myeloid cells.

A total of 4279 B cells were analyzed and classified into five subtypes and Bcell_C3 was annotated as plasma cells using the inner databases of singleR (Figure 5A, Supporting Information Figures S6A,B,E & S7A). The relative abundance of the B‐cell subtype showed no uniform pattern in different patient foci and anatomical pathology (Figure 5C, Supporting Information Figure S6C,D). The pathway analysis of B cells further showed that there were two types of B cells with different functions in MPLC. The regulation and activation of the innate immune response were highly activated in Bcell‐C3 and Bcell‐C5 clusters, whereas Bcell‐C1, Bcell‐C2 and Bcell‐C4 clusters were enriched in pathways related to antigen binding and processing and presentation (Figure 5D). The results of trajectory analysis indicated that Bcells_C1 had two differentiation states: one may differentiate into Bcell_C3 and Bcell_C5 and the other may differentiate into Bcell_C4 (Figure 5B, Supporting Information Figure S7B‐D). In addition, different from TCR clonotypes sharing more overlap and correlation in foci grouped according to the source within the patient, the BCR clonotypes shared little overlap in MPLC (Supporting Information Figure S6F).

### 3.6 Myeloid cells have two functional distribution subtypes in MPLC

A total of 44 261 myeloid cells were subclustered into seven subsets (Figure 5E, Supporting Information Figure S9A,D). The percentage of M2 macrophages increased from normal to IAC, while other subtypes were not significantly different between MIA/AIS and IAC (Figure 5F, Supporting Information Figure S9B). Macrophages contribute significantly to organismal integrity by either repairing tissue under sterile inflammatory settings or engaging in pathogen elimination.^40^ From a functional perspective, myeloid cells were divided into two main types: M1 macrophages can positively regulate the innate immune response and M2 macrophages could negatively regulate the adaptive immune response and regulation of stem cell differentiation. Lan et al. showed that M2 macrophages within the TME played an important role in facilitating tumour migration and invasion,^41^ and our findings implied that the increase in M2 macrophages would lead to malignant cells adapting to the immune response and escaping the response from the immune system (Supporting Information Figure S9C).

The results of trajectory analysis indicated that myeloid cells had four main branch trajectories (Supporting Information Figure 5H). M2 macrophages were mainly concentrated in the left branch trajectory, whereas M1 macrophages were mainly in the opposite branch trajectory (Figure 5H). Next, we evaluated the expression of differentially expressed genes (DEGs) in the M1 macrophage and M2 macrophage populations in the whole trajectory. The expression of macrophage colony‐stimulating factor genes increased gradually during the process of transdifferentiating into M1 macrophages, and the expression of genes related to cytomembrane transport increased during the process of transdifferentiating into M2 macrophages (Supporting Information Figure S9F).

### 3.7 Positive regulation of endothelial cell development is extensive in IAC

**FIGURE 6:**
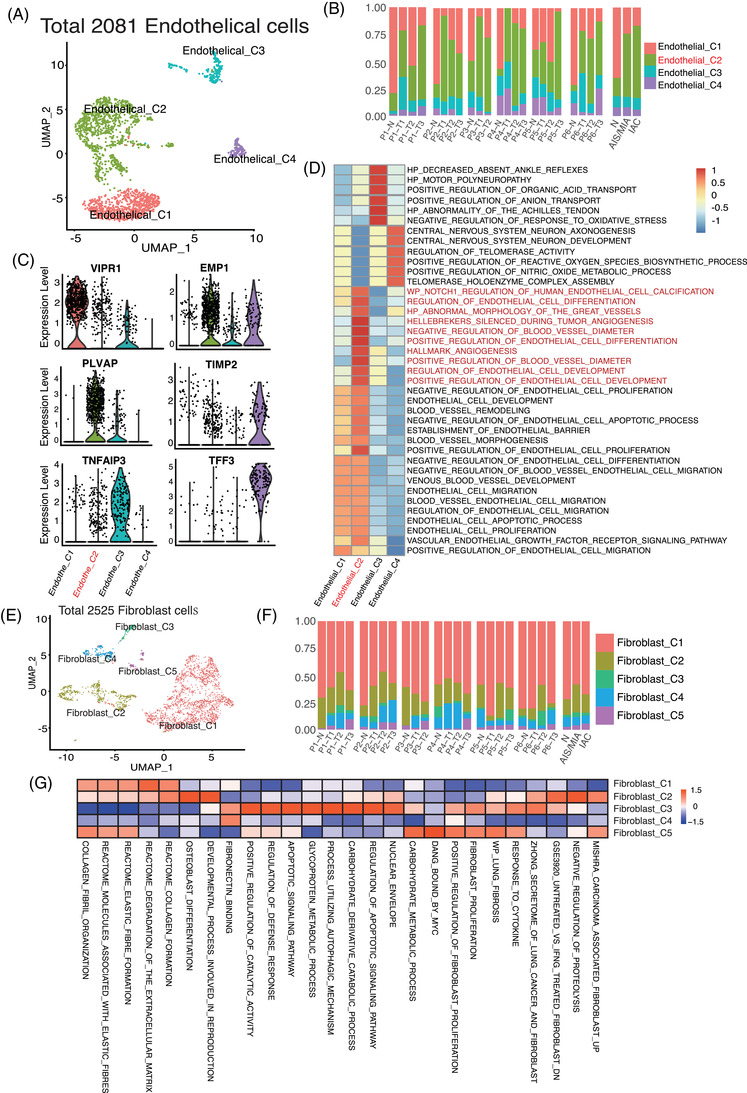
Endothelial cell and fibroblast distributions in MPLC. (A) UMAP projection of 2081 endothelial cells showing the composition of the four main subtypes. (B) Percentages of each endothelial cell subtype among normal, AIS/MIA and IAC samples. *Y* axis: Average percentage of samples across the three groups. Groups are separated by blank space. Each bar plot represents different samples and the last bar plot is grouped by sample type (normal, AIS/MIA and IAC). (C) Violin plot of marker gene expression in Endothelial_C2 cell clusters. (D) Differentially expressed pathways were scored per cell by GSVA between the four endothelial cell subtypes. (E) UMAP projection of 2525 fibroblasts showing the composition of the five main subtypes. (F) Percentages of each fibroblast subtype among normal, AIS/MIA and IAC samples. (G) Differentially expressed pathways were scored per cell by GSVA between the five fibroblast subtypes.

We found four distinct subtypes by clustering 2081 endothelial cells (Figure 6A C, Supporting Information Figure S8A,D). Compared with normal tissues, Endothelial_C2 was significantly abundant in IAC and an increasing trend was observed from normal samples to IAC. But differences were not obvious for other types of endothelial cells among normal samples, AIS/MIA and IAC (Figure 6B, Supporting Information Figure S8C). Herein, we focused on the transcriptomic features and functions of Endothelial_C2 cells, and the marker genes of Endothelial_C2 are presented in Figure 6C. The pathway analyses showed that Endothelial_C2 cells were associated with regulation of endothelial cell development and differentiation (Figure 6D).

### 3.8 Fibroblast subtype distributions are similar in IAC, AIS/MIA and normal samples

Fibroblasts maintained a very stable state in MPLC and a total of 2523 fibroblasts were clustered into five subtypes (Figure 6E, Supporting Information Figure S8E). In MPLC, the abundance of these five fibroblast clusters showed insignificant differences among normal, AIS/MIA and IAC samples (Figure 6F, Supporting Information Figure S8F). According to the results of pathway analysis, different pathways were found for each type of fibroblasts (Figure 6G). Fibroblast_C1 cells expressed high levels of genes associated with the Reactome elastic fibre formation annotation, whereas Fibroblast_C5 cells highly expressed genes related to positive regulation of fibroblast proliferation (Figure 6G, Supporting Information Figure S8G).

### 3.9 Characterization of cell–cell interactions involved in MPLC

**FIGURE 7:**
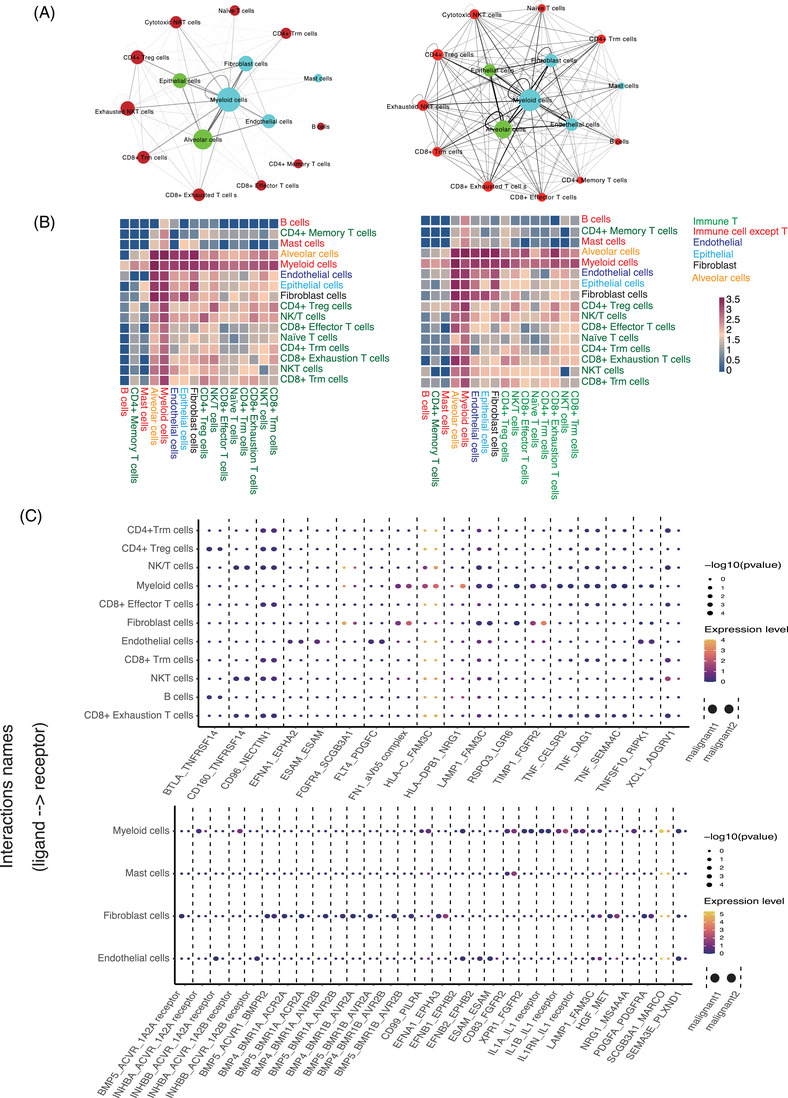
Inter‐cellular interactions in MIA lung tumours and IAC lung tumours. We quantified the predicted cell interactions in MIA and IAC lung cancer. (A) Networks depicting cell types as nodes and interactions as edges. The size of the node is proportional to the total number of interactions of the corresponding cell type, and edge thickness is proportional to the number of interactions between the connected types. Left is MIA and right is IAC. (B) Heat map depicting the number of all possible interactions between the clusters analyzed. Different cell types are identified by different colours. Left is MIA and right is IAC. (C) Dot plot depicting selected ligand–receptor interactions between two states of epithelial cells and other cells in MPLC.

Compared with the MIA samples, we found that cells in IAC samples had significantly increased interactions (Figure 7A). Alveolar and myeloid cells had the most significant interactions with all cell types in both MIA and IAC samples. In comparison with MIA, the interaction between T cells and alveolar cells was significantly increased in IAC samples, indicating the active roles of these two cell types in malignant samples (Figure 7B). Myeloid, cytotoxic T, natural killer and B cells might be involved in the action of recognizing and presenting the antigens of epithelial cells among the interactions from immune cells to epithelial cells. Next, we analyzed the ligand‐receptor interactions between two states of malignant cells (Malignant1 and Malignant2) and other immune cells. Most of the ligand‐receptor interactions were similar for these two types of malignant cells, while some interactions between immune cells and each type of malignant cell were distinct (Figure 7C). As for the ligand‐receptor interactions of three different types of samples, our findings showed that interactions related to immune checkpoint axis (HLA‐E_KLRK1) were significantly more abundant in IAC than in AIS/MIA and normal samples (Supporting Information Figure S10A, upper; b). Cellular adhesion is regarded as a crucial hallmark in cancer, and the molecules participating in cell‐to‐cell adhesion are usually deregulated in cancer progression and metastasis.^42^ The interactions related to independent cellular adhesion (NECTIN1_CADM3, FGF1_FGFR4 and CD44_FGFR2) seemed to be less abundant in epithelial cells than in fibroblasts, implying that epithelial cells might promote structural changes in fibroblasts and reduce cell adhesion (Supporting Information Figure S10A, middle). On the one hand, epithelial cells may reduce fibroblast adhesion; on the other hand, they may strengthen the differentiation of other mesenchymal cells with deeper invasion in MPLC. Previous studies have revealed that cancer cells may activate the PI3K/Akt signalling pathway and promote endothelial cell tube formation, suggesting the critical roles of endothelial cells in angiogenesis during cancer progression.^43^ According to our results, the interactions related to endothelial and hematopoietic stem cell differentiation and development (SELL‐CD34, SELP‐CD34, NPR1‐NPPC and NPR2‐NPPC) seemed to be more abundant in IAC than in normal and AIS/MIA samples (Supporting Information Figure S10A, down).

## 4 DISCUSSION

MPLC is becoming more popular due to the widespread application of early detection methods.^44^ Emerging evidence revealed the molecular characteristics and cellular interactions of NSCLC using scRNA‐seq.^45^, ^46^, ^47^ For example, Peng et al. have shown that N1‐like neutrophils could enhance the activation of T and B cells in NSCLC, while nearby M2‐like macrophages exhibited inhibitory effects.^46^ Leader et al. have revealed that SPP1^+^ macrophages and PDCD1^+^CXCL13^+^ activated T cells were correlated with the activation of lung cancer, indicating their essential role in cancer progression.^47^ Furthermore, HK2^+^/CK^−^‐circulating tumour cells exhibited poor responses to treatment and were related to unfavourable clinical outcomes in patients with lung cancer.^48^, ^49^ However, the comprehensive cellular characteristics and evolution of different cell types during the development of MPLC remain unclear. Few studies have thoroughly investigated the cellular landscape of different sites of MPLC. In this study, we comprehensively evaluated changes in cellular features and inter‐cellular connections during the progression from normal tissue to IAC sample in MPLC, which may provide novel insight into the mechanism of tumour progression and specific treatment for MPLC.

Recent studies have performed similar development trajectory analyses for CD8^+^ T cells, which revealed that the root was naïve T cells and the two‐end states were exhausted T cells and cytotoxic T cells.^24^, ^50^, ^51^ However, naïve T cells were not the only root for CD8^+^ T‐cell development and evolution in MPLC. We found that memory T cells were also a main source of these cells and played an important role in CD8^+^ T‐cell differentiation. Pathways correlated with immune response were significantly enriched during this differentiation process. However, compared with CD8^+^ memory T cells, the progression of CD8^+^ naïve T cells to effector T cells was mainly a process of leukocyte activation and T‐cell differentiation. Moreover, effector T cells might differentiate into exhausted T cells, which involve responses to stimuli and positive regulation of cell death. As for the CD4^+^ T cells, two distinct functional states (Treg and naïve cells) were identified, and eight subclusters were subsequently identified. According to the results of pathway analyses, each subcluster had a distinct function, suggesting their different roles in the development and progression of MPLC. We also found that T cells shared more consistent TCR clonotypes than AIS/MIA‐group cells in normal and IAC samples. Unfortunately, further in‐depth study of this interesting phenomenon was beyond the scope of this study.

In contrast to previous studies on the metabolism of malignant cells,^24^, ^52^, ^53^, ^54^ we used trajectory analysis to study the transition of epithelial cells from normal to malignant. Although different nodules in the patients were heterogeneous, they all exhibited two states during the evolution of malignant cells from AIS/MIA to IAC. Specifically, malignant_1 cells were closely related to the response of immune system, while malignant_2 cells underwent programmed cell death or entered the cell cycle due to selective pressure from the development of tumours in MPLC. Our findings indicated that malignant_1 cells may serve a vital role in tumour progression, which might be the potential therapeutic target in MPLC. Considering the close relationship between malignant_1 cells and immune system process, immunotherapy could also be an option for patients with MPLC. In addition, the proportion of AT2 cells gradually decreased with the development of LUAD from normal to AIS/MIA to IAC, which also provided evidence that AT2 was the most likely origin of malignant cells in MPLC.^39^ Due to the limited number of patients enrolled in our study, more patients need to be enrolled to explore and validate the function of AT2 in lung cancer.

Our findings indicated that the functional distribution subtypes of B cells can be distinguished into two different subpopulations. One was closely related to the regulation and activation of innate immune response, and the other was correlated with antigen processing and presentation including MHC class II antigen presentation. We found that these two distinct subpopulations of B cells participated in different processes of immune responses, indicating the various functions of B cells within the TME of MPLC. Myeloid cells were divided into two major types: one was correlated with positive regulation of the innate immune response, whereas the other performed negative regulation of the adaptive immune response and stem cell differentiation presented by M2 macrophages. With the progression of LUAD from normal to IAC, the increasing proportion of M2 macrophages caused malignant cells to adapt to the immune response and escape from the immune response.^55^, ^56^, ^57^ And we have identified two main types of macrophages in MPLC, which were similar to previous studies. In addition, Endothelial_C2 participated in the positive regulation of blood vessel development in early glandular neoplasia of the lung, suggesting the potential role of this specific endothelial cell cluster in the development and progression of MPLC. Moreover, compared with other subclusters of endothelial cells, the proportion of Endothelial_C2 gradually increased with the progression of LUAD from normal to IAC.^58^, ^59^, ^60^, ^61^ And Endothelial_C2 might play a significant role in the progression of LUAD in MPLC patients, which may be a potential target for cancer early detection and treatment.

A comprehensive analysis of cell–cell interactions in MPLC revealed that inter‐cellular interactions may be more complex in IAC than in MIA, indicating that cells and their related communications may increase with the malignant degree of tumour. Furthermore, interactions related to independent cellular adhesion seemed to be less abundant in epithelial cells than in fibroblasts. The interactions related to endothelial and haematopoietic stem cell differentiation and development seemed to be more abundant in the IAC than in the normal and AIS/MIA samples. Our findings suggested that the inter‐cellular interactions were distinct among different samples of MPLC, which may be the key mechanism in the development of MPLC. Besides, some distinct ligand–receptor interactions of each malignant cell should be further analyzed in future research.

Despite the striking findings presented in this study, there were still several limitations, including sample collection, technical challenges and software limitations. First, we were unable to evaluate the continuous development of different nodules in the same patient, and the cells used for single‐cell sequencing were from just a part of the tumour tissue. There might be differences in biological complexity between our samples and the tissues used for pathological diagnosis. Second, the 10× Genomics single‐cell technology cannot cover all the transcripts in cells. Moreover, there were also some limitations in batch effect removal, clustering and trajectory inference analysis, as these analyses were still maturing, and the complexity of the underlying topology could be underestimated.^62^

## 5 CONCLUSIONS

In the present study, we have used scRNA‐seq and WES techniques to comprehensively evaluate the genomic and cellular features of various sites in MPLC patients. Our findings revealed the dynamic changes of different cellular compositions from normal samples to AIS/MIA and finally to IAC. We have identified two states of malignant cells with different functions, and identified various subpopulations of immune and stromal cells in MPLC patients, which may provide novel insight into the development of effective therapeutic strategies.

## CONFLICT OF INTEREST STATEMENT

The authors report no conflict of interest.

## FUNDING INFORMATION

This study was supported by the National Key R&D Program of China (2021YFC2500900), the National Natural Science Foundation of China (82002451), the CAMS Initiative for Innovative Medicine (2021‐1‐I2M‐015) and the Beijing Hope Run Special Fund of Cancer Foundation of China (LC2019B15).

## ETHICS APPROVAL

The Ethics Committee and Institutional Review Boards of Cancer Hospital, Chinese Academy of Medical Sciences and Peking Union Medical College approved this study, and the signed informed consents were obtained from all patients (Approval number: NCC2208).

## Supporting information

Supporting Information

Supporting Information

Supporting Information

Supporting Information

## Data Availability

The raw data that support the findings of this study are available from the corresponding author upon reasonable request.
