# Supplementary material for: Single‐cell RNA sequencing analysis reveals transcriptional heterogeneity of multiple primary lung cancer
Source: Clin Transl Med. 2023 Oct 17;13(10):e1453. doi: 10.1002/ctm2.1453 (PMC10580343; doi:10.1002/ctm2.1453)
Supplement: Supplementary file 1 — Supporting Information [file CTM2-13-e1453-s003.docx]

Supplemental Figures

Supplemental Figure 1


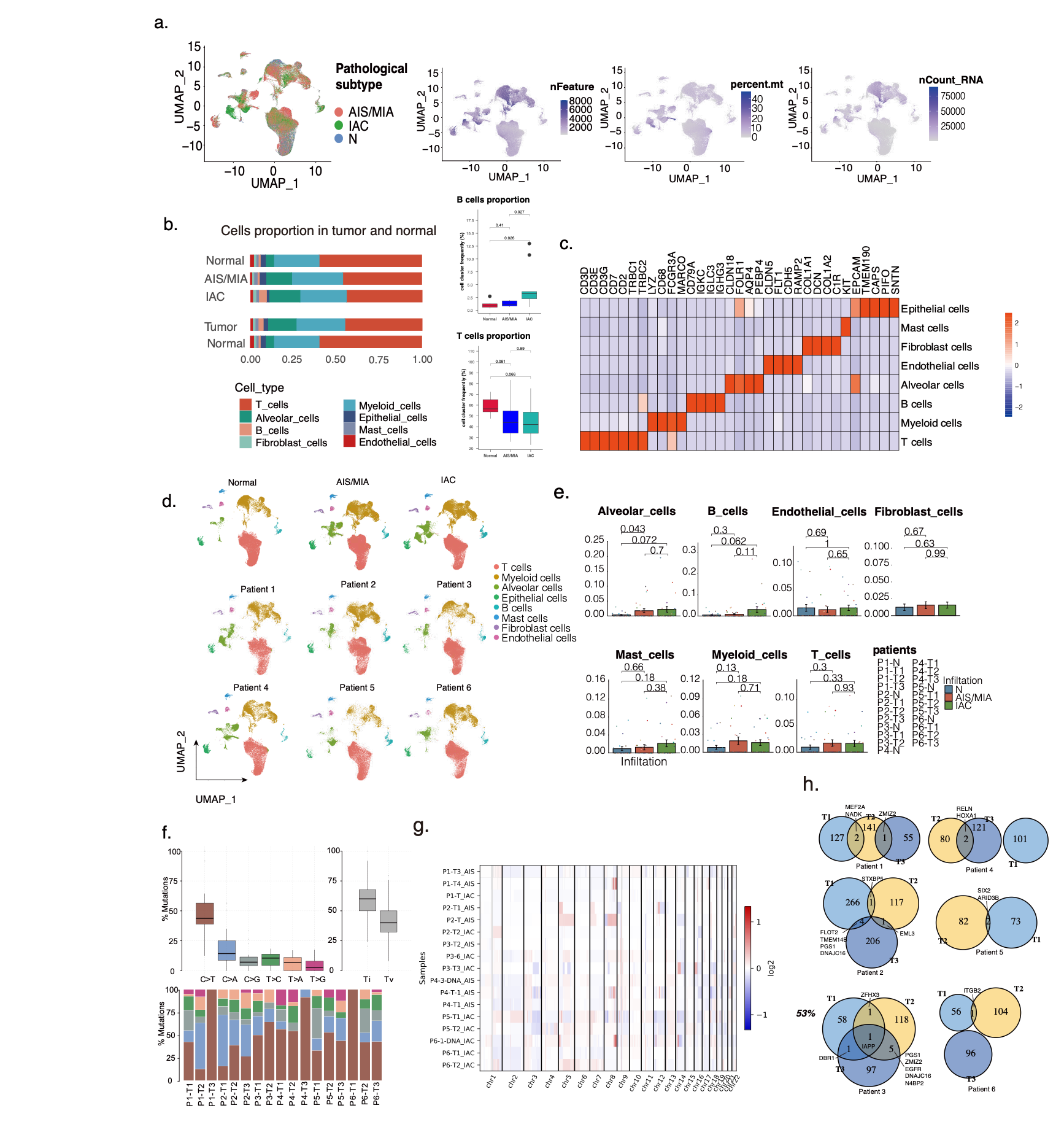


**Supplement Fig.1 a.** Uniform Manifold Approximation and Projection (UMAP) plot of 167,397 cells, color-coded by anatomical pathology info (left), expressing gene number (mid1), percentage of expressing mitochondrial gene (mid2), Transcript number (right) in the synchronous multiple primary lung cancer dataset. **b.** Proportions of the global cell types in synchronous multiple primary lung cancer tissue and normal in samples (left). Box plots showing the relative proportion of B and T cells in different subgroups (right). **c.** The heatmap of Marker Genes which were used to label clusters by cell identity as represented in the UMAP plot. **d.** Uniform Manifold Approximation and Projection (UMAP) plot color-coded by normal, AIS/MIA, IAC and each patients info. **e.** Average proportion of all kinds of cells among all nLung, AIS/MIA, and IAC samples. **f.** Mutational landscape of MPLC. Box-plot of SNV class (Upper in f). Proportions of the mutation in synchronous multiple primary lung cancer tissue by individual samples (Down in f). g. The heatmap showing the differences of CNV in different lesions. h. The overlap and distinct mutations in different lesions of each MPLC patient.

Supplemental Figure 2

**Supplement Fig.2 a.** UMAP plot of all epithelial cells of the patients, color-coded by anatomical pathology info (left), colored according to patient info(right).**b** Relative proportions of all epithelial cells subpopulations grouped by anatomical pathology info and Tumor info. **c** UMAP plot of all epithelial cells of the patients, color-coded by number of genes(left), percentage of expressing mitochondrial genes(Middle), Transcript number(Right). **d** The balloon plot of marker genes in the subtype of epithelial cells. **e** Heatmap showing large-scale CNVs for individual cells (rows). Nonmalignant cells (Immune cells included T cells and B cells)were treated as references (down), and large-scale CNVs were observed in malignant cells (upper). The color shows the log2 CNV ratio. Red: amplifications; blue: deletions. f. Unsupervised pseudotime trajectory analysis of all epithelial cells by Monocle 3, visualizing TMB info. **g** Heatmap showing scaled expression of dynamic genes along the pseudotime. Rows of the heatmap represent genes that show dynamic changes along the pseudotime, and these genes were clustered into two groups according to their expression pattern along the pseudotime.

Supplemental Figure 3


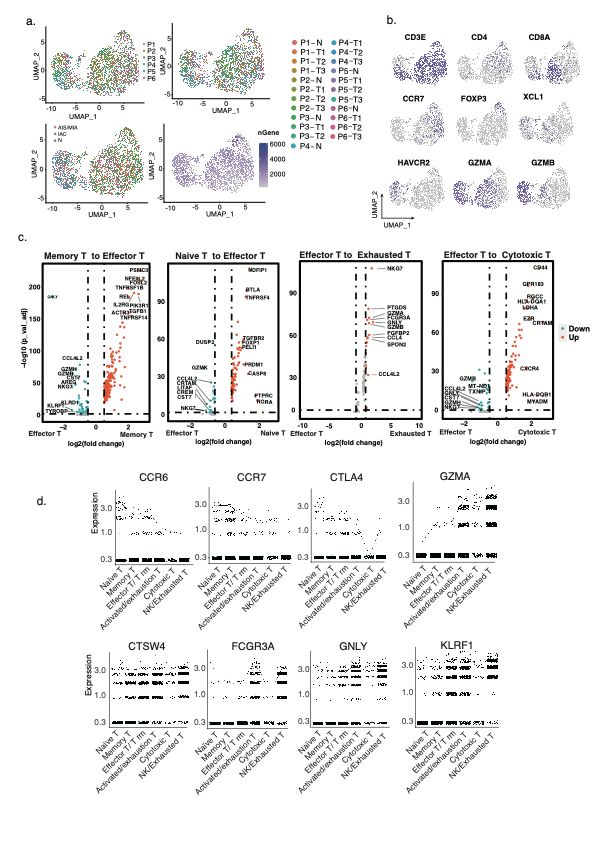


**Supplement Fig.3 a** Uniform Manifold Approximation and Projection (UMAP) plot of total 81171 T cells colored according to patient info, anatomical pathology info and gene number. b. Canonical marker genes were used to label T cells subtypes as represented in the UMAP plot. **c.** The volcano plot that expression difference genes comparing the expression of genes in the next stage cells(right) to those in the previous stage(left),followed by Memory T to effector T, naïve T to effector T cells, Effector T transforming to Exhausted T cells, effector to cytotoxic T cells. **d.** Spline curve fitted to the expression of DEGs in CD8+ Tcells along the total pseudotime trajectory.

Supplemental Figure 4

**Supplement Fig.4 a** Heatmap showing the fraction of shared clonotypes belonging to a primary phenotype cluster (rows) that are shared with other secondary phenotype clusters (columns) followed by all kinds of T cells to all kinds of T cells(Left-Up), T cells in IAC to T cells in AIS/MIA(Right-up), T cells in Normal to AIS/MIA, T cells in Normal tp IAC. **b** Bar plot of T cell cluster assignments for the top 20 TCR specificity groups, separated by *TRB-TRA* clone, grouped by Normal(left), AIS/MIA(middle) and IAC(right).

Supplemental Figure 5

**Supplement Fig.5 a.** The marker genes of each CD4^+^ T cell and the relevant UMAP plot. **b.** Violin plots depicting the expression of immune genes in the CD4^+^ T cell divided by tissue of origin**.** c. The heatmap of differential expressed pathways are scored by GSVA grouped by CD4+ Naïve T cells subtypes (Left) and CD4+ Treg cells subtypes(Right).

Supplemental Figure 6

**Supplement Fig.6 a.**UMAP plot of B cells colored according to patient info, sample info , anatomical pathology info, gene numbers, percentage of expressing mitochondrial genes . **b.** UMAP plot of B cells color-coded by the anatomical pathology info that Normal, AIS/MIA, IAC separately. **c.** Relative proportion of cells contained in each sample in different B cell subtypes. **d.** Average proportion of all kinds of B cells among all nLung, AIS/MIA, and IAC samples. **e.** Canonical marker genes were used to label B cells subtypes as represented in the UMAP plot. **f.** The Correlation of the BCR Clonetypes in different samples(Left). Each tile represents the correlation of coefficient of clones. Color intensity indicates correlation ratio. The Overlap of the BCR Clonotypes in each sample(Right). Each tile represents the overlap of coefficient of clones. Color intensity indicates overlap strength.

Supplemental Figure 7

**Supplement Fig.7 a.** Heatmap of marker gene expression in B cells clusters. **b.** Unsupervised pseudotime trajectory analysis of B cells color-coded by different B cell subtypes. **c**. Heatmap showing scaled expression of dynamic genes along the pseudotime from Bcell_c1 differentiate into Bcell_C4 and Bcell_C3. Rows of the heatmap represent genes that show dynamic changes along the pseudotime, and these genes were clustered into three groups according to their expression pattern along the pseudotime. **d**. Spline curve fitted to the expression of DEGs in B cells along the total pseudotime trajectory.

Supplemental Figure 8

**Supplement Fig.8 a**. UMAP plot of endothelial cells colored according to patient info(Upper), sample info (Middle), anatomical pathology info(Down). **b.** Relative proportion of cells contained in each sample in different ecell subtypes. **c.** Average proportion of all kinds of endothelial cells among all nLung, AIS/MIA, and IAC samples. **d**. Heatmap of marker gene expression in endothelial cells clusters. **e.** UMAP plot of fibroblast cells colored according to patient info(Upper), anatomical pathology info (Middle), sample info (Down). **f.** Average proportion of all kinds of fibroblast cells among all nLung, AIS/MIA, and IAC samples. **g.** Heatmap of marker gene expression in fibroblast cells clusters.

Supplemental Figure 9


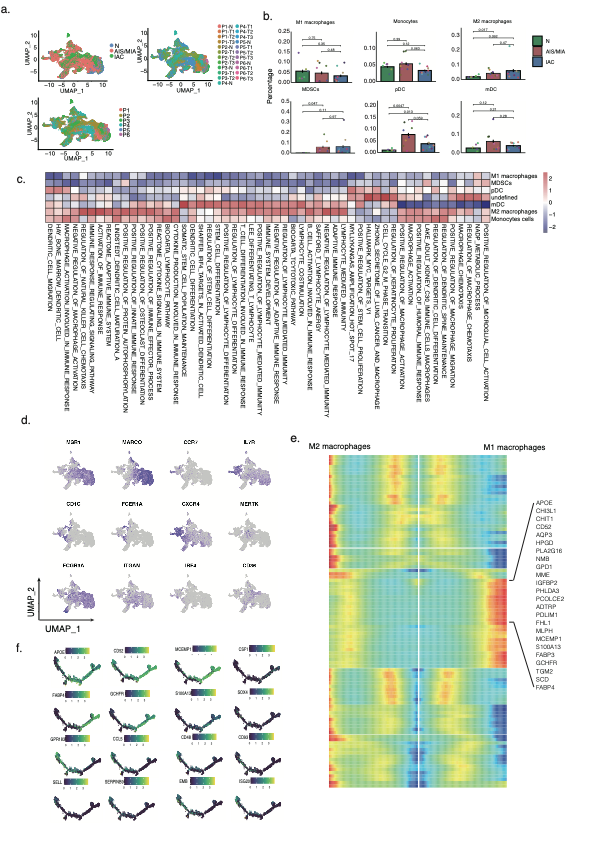


**Supplement Fig.9 a.** UMAP plot of myeloid cells colored according to patient info(Left-down), sample info (Right-up), anatomical pathology info(Left-up). **b.** Average proportion of all kinds of myeloid cells among all nLung, AIS/MIA, and IAC samples. **c**. The heatmap of differential expressed pathways are scored by GSVA grouped by myeloid cells subtypes. **d.** Canonical marker genes were used to label myeloid cells subtypes as represented in the UMAP plot. **e.** Heatmap showing scaled expression of dynamic genes along the pseudotime from myeloid cells differentiate into M1 macrophages and M2 macrophages. Rows of the heatmap represent genes that show dynamic changes along the pseudotime, and these genes were clustered into three groups according to their expression pattern along the pseudotime. **f.** Spline curve fitted to the expression of DEGs in myeloid cells along the total pseudotime trajectory.

Supplemental Figure 10

**Supplement Fig.10 a.** Dot plot depicting selected immune–epithelial, epithelial–mesenchymal and mesenchymal–endothelial interactions enriched in MPLC (left to right: Normal, AIS/MIA, IAC). **b.** Boxplot comparing the mean value of ligand-recepotor interactions between IAC and AIS/MIA.
