# Supplementary material for: Single‐cell RNA sequencing analysis reveals transcriptional heterogeneity of multiple primary lung cancer
Source: Clin Transl Med. 2023 Oct 17;13(10):e1453. doi: 10.1002/ctm2.1453 (PMC10580343; doi:10.1002/ctm2.1453)
Supplement: Supplementary file 2 — Supporting Information [file CTM2-13-e1453-s001.docx]

| Supplementary Table 1. The clinical and specimen information of patients in this study. | | | | | | | | | | | |
| --- | --- | --- | --- | --- | --- | --- | --- | --- | --- | --- | --- |
| Patient_number | Sample_number | Specimen species | Age | Gender | Smoking history | Family history of lung cancer | Tumor size (cm) | Radiological feature | Location | Histology | Lymph node metastasis |
| Patient 1 | P1-N | adjacent tissues | 56 | Male | Yes | Yes |  |  |  | Normal | No |
|  | P1-T1 | tumor tissues |  |  |  |  | 1.4 | GGO | RLL | MIA |  |
|  | P1-T2 | tumor tissues |  |  |  |  | 1 | GGO | RUL | MIA |  |
|  | P1-T3 | tumor tissues |  |  |  |  | 1.6 | Subsolid | LLL | IAC |  |
| Patient 2 | P2-N | adjacent tissues | 68 | Male | Yes | No |  |  |  | Normal | No |
|  | P2-T1 | tumor tissues |  |  |  |  | 2.2 | GGO | RUL | IAC |  |
|  | P2-T2 | tumor tissues |  |  |  |  | 1.5 | GGO | RML | MIA |  |
|  | P2-T3 | tumor tissues |  |  |  |  | 2.2 | GGO | LUL | MIA |  |
| Patient 3 | P3-N | adjacent tissues | 52 | Female | No | No |  |  |  | Normal | Yes |
|  | P3-T1 | tumor tissues |  |  |  |  | 1.7 | Solid | RUL | IAC |  |
|  | P3-T2 | tumor tissues |  |  |  |  | 1.2 | Subsolid | RLL | IAC |  |
| Patient 4 | P4-N | adjacent tissues | 49 | Female | No | No |  |  |  | Normal | No |
|  | P4-T1 | tumor tissues |  |  |  |  | 1.8 | Subsolid | RUL | MIA |  |
|  | P4-T2 | tumor tissues |  |  |  |  | 1 | Subsolid | LLL | MIA |  |
|  | P4-T3 | tumor tissues |  |  |  |  | 1.2 | GGO | LLL | MIA |  |
| Patient 5 | P5-N | adjacent tissues | 66 | Male | No | No |  |  |  | Normal | No |
|  | P5-T1 | tumor tissues |  |  |  |  | 1 | GGO | RUL | AIS |  |
|  | P5-T2 | tumor tissues |  |  |  |  | 1.6 | Solid | RUL | IAC |  |
|  | P5-T3 | tumor tissues |  |  |  |  | 1.9 | Subsolid | LUL | IAC |  |
| Patient 6 | P6-N | adjacent tissues | 63 | Female | No | No |  |  |  | Normal | No |
|  | P6-T1 | tumor tissues |  |  |  |  | 2.9 | Subsolid | RUL | IAC |  |
|  | P6-T2 | tumor tissues |  |  |  |  | 1 | GGO | RLL | IAC |  |
|  | P6-T3 | tumor tissues |  |  |  |  | 0.9 | Subsolid | LUL | IAC |  |
