# Supplementary material for: Single‐cell RNA sequencing analysis reveals transcriptional heterogeneity of multiple primary lung cancer
Source: Clin Transl Med. 2023 Oct 17;13(10):e1453. doi: 10.1002/ctm2.1453 (PMC10580343; doi:10.1002/ctm2.1453)
Supplement: Supplementary file 3 — Supporting Information [file CTM2-13-e1453-s004.docx]

| Supplementary table2. Epithelial cell information of the 6 MPLC patients' samples | | | | | |  |
| --- | --- | --- | --- | --- | --- | --- |
|  | AT1 | AT2 | Ciliated | Club | malignant cells | Others |
| P1-N | 41 | 8 | 195 | 59 | 0 | 89 |
| P1-T1 | 9 | 27 | 171 | 22 | 874 | 0 |
| P1-T2 | 13 | 137 | 121 | 5 | 403 | 0 |
| P1-T3 | 46 | 69 | 425 | 55 | 4139 | 0 |
| P2-N | 196 | 7 | 360 | 51 | 0 | 443 |
| P2-T1 | 4 | 445 | 166 | 46 | 53 | 0 |
| P2-T2 | 83 | 465 | 698 | 83 | 255 | 0 |
| P2-T3 | 6 | 219 | 189 | 15 | 116 | 1 |
| P3-N | 77 | 3 | 152 | 17 | 0 | 155 |
| P3-T1 | 14 | 7 | 108 | 13 | 115 | 0 |
| P3-T2 | 33 | 20 | 251 | 8 | 2354 | 1 |
| P4-N | 115 | 12 | 255 | 96 | 0 | 140 |
| P4-T1 | 4 | 12 | 260 | 47 | 1951 | 176 |
| P4-T2 | 60 | 1572 | 437 | 57 | 102 | 0 |
| P4-T3 | 33 | 1557 | 921 | 116 | 154 | 1 |
| P5-N | 35 | 13 | 71 | 19 | 0 | 97 |
| P5-T1 | 32 | 53 | 129 | 28 | 202 | 0 |
| P5-T2 | 21 | 47 | 84 | 31 | 167 | 0 |
| P5-T3 | 29 | 356 | 131 | 21 | 517 | 0 |
| P6-N | 32 | 6 | 180 | 48 | 0 | 63 |
| P6-T1 | 5 | 8 | 144 | 28 | 244 | 1 |
| P6-T2 | 18 | 155 | 276 | 54 | 275 | 4 |
| P6-T3 | 5 | 21 | 215 | 4 | 1645 | 0 |
